# Large-scale production of *Mansonella perstans* infective larvae from engorged *Culicoides milnei*

**DOI:** 10.3389/fitd.2024.1391823

**Published:** 2024-12-10

**Authors:** Chi Anizette Kien, Rene Ebai, Fanny Fri Fombad, Frederick Esofi, Anna Ning Ntuh, Emmanuel Ouam, Narcisse Victor Tchamatchoua Gandjui, Valerine Chawa Chunda, Relindis Ekanya, Franck Noel Nietcho, Juluis Visnel Foyet, Lucy Cho Nchang, Chefor Magha, Abdel Jelil Njouendou, Peter Enyong, Achim Hoerauf, Manuel Ritter, Samuel Wanji

**Affiliations:** 1 Parasite and Vector Research Unit (PAVRU), Department of Microbiology and Parasitology, University of Buea, Buea, Cameroon; 2 Research Foundation for Tropical Diseases and the Environment (REFOTDE), Buea, Cameroon; 3 Institute for Medical Microbiology, Immunology and Parasitology (IMMIP), University Hospital Bonn (UKB), Bonn, Germany; 4 German-West African Centre for Global Health and Pandemic Prevention (G-WAC), Partner Site Bonn, Bonn, Germany; 5 German Centre for Infection Research (DZIF), Bonn-Cologne partner site, Bonn, Germany

**Keywords:** *Mansonella perstans*, infective stage 3 larvae (L3), *Culicoides milnei*, midges, vector abundance, survival and mortality of *Culicoides* species

## Abstract

**Background:**

*Mansonella perstans* is transmitted by *Culicoides* species and affects hundred millions of inhabitants in about 33 countries in sub-Saharan Africa. It is known that Mansonellosis due to *Mansonella perstans* do not result in a clear clinical picture, but down-regulates the immunity of patients predisposing them to other diseases like tuberculosis, HIV and malaria or damping vaccine efficacy. However, research about novel drugs against this filarial nematode is missing because of the lack of parasite material. Previous studies have developed *in vitro* culture systems using infective stage 3 larvae (L3), but these life stages are difficult to obtain and thus the performance of *in vitro* cultures is restricted and does not allow large-scale testing of drugs or even infection experiments in animal models. Therefore, we aim to establish a platform for the large-scale production of *M. perstans* infective larvae from engorged *Culicoides milnei*.

**Methods:**

*Culicoides* species were caught in Yangom (Yabassi Health District) in the Littoral Region of Cameroon following a blood meal on six microfilariae-positive donors with different microfilaraemic loads over one year. Engorged midges were reared in the insectarium for up to 14 days and L3 were isolated from the different body parts.

**Result:**

In summary, 13,658 engorged *Culicoides* were collected and reared in the laboratory. We observed an overall predicted survival of 78.5%. Out of the 8,123 survived midges, 7,086 midges belong to *C. milnei*, from which 2,335 were infected leading to a recovery of 6,310 L3. Moreover, we found the highest survival rates of midges during the early dry season in December with moderate temperatures (23-25°C) and low (2-4mm) or no rainfall. In addition, we observed that midges that fed on donors with high microfilarial loads showed increased mortality.

**Conclusion:**

We revealed suitable conditions for the collection and maintenance of engorged *Culicoides* midges allowing the large-scale production of *M. perstans* L3. This procedure will provide a platform to produce sufficient parasite material that will facilitate *in vitro* cultures and the establishment of a murine model of *M. perstans*, which is important for in-depth investigation of the filarial biology and screening of novel drugs that are effective against this ivermectin-resistant nematode.

## 1 Introduction

Mansonellosis is caused by *Mansonella perstans, M. ozzardi, M. streptocerca* and *M*. sp. *DEUX* (1, 2), but zoonotic species like *M. rhodhaini* have been described as well (3). In contrast to lymphatic filariasis or onchocerciasis, no severe clinical symptoms are associated with mansonellosis, but subcutaneous swellings, skin rashes, pericarditis and pleuritis, keratitis and eosinophilia have been reported (4). It is suggested that the lack of clinical symptoms is associated with a strong immunomodulatory capacity (2, 5), which has led to a shortfall of knowledge about this parasite, but is also associated with increased susceptibility and worsened disease course of concomitant infections (6–8) accompanied with lowered vaccination efficacy (9). Thus, knowledge about this filarial nematode, its biology and evasion tactics are needed to develop treatment strategies. Most of the knowledge about mansonellosis is obtained from *M. perstans* infections, which is widespread in Sub-Saharan Africa, Central and South America and was even considered as the most prevalent parasite of man in tropical Africa (10). In spite of this, very few studies have carried out its epidemiology and the associated health consequences in endemic populations, and no simple and effective drug therapy for treatment and control of the infection has been identified. A study in Cameroon revealed that *M. perstans* is mainly prevalent in equatorial rainforest and that ivermectin mass drug administration is not effective (11), whereas doxycycline can reduce microfilariae (mf; the worm’s offspring) in the peripheral blood (12, 13) confirming that *M. perstans* harbors *Wolbachia* endosymbionts (14, 15).

*M. perstans* is transmitted by *Culicoides* and their abundance and seasonal occurrence depends on habitats close to streams, ponds and marshes (16, 17). Recently, we revealed that *C. milnei*, a nocturnal species, is the major vector of *M. perstans* in the South-West and Littoral regions of Cameroon (18, 19), which transmits infective larvae (L3) that develop into adult worms which reside in body cavities like peritoneum, pleura and pericardium. Thus, adult worms have only been recovered on rare occasions and consequently research about the development of the different life stages remains limited. Therefore, *in vitro* cultures provide a crucial tool to unravel many open questions about this filarial nematode. Indeed, we established *in vitro* cultures of *M. perstans* microfilariae and adult worms to study the biology of the filarial nematode and test anti-filarial drugs (20–22). However, the implementation of *in vitro* culture depends on the collection of engorged *Culicoides* and the isolation of infective larvae, which is a sophisticated and difficult process, since it requires knowledge about the vector and its biting behavior, techniques to collect and maintain engorged midges and isolate L3 from the different body parts. To overcome this issue, we established and optimized the procedure implemented from our previous publications (20, 22) to scale up the production of *M. perstans* infective larvae, which will facilitate *in vitro* cultures and large-scale drug testing approaches as well as provide a platform for the implementation of murine *M. perstans* infection models.

## 2 Methods

### 2.1 Ethics

Ethical clearance was obtained from the Cameroon National Ethics Committee, Yaoundé (REF: N° 2022/12/1506/CE/CNERSH/SP) and administrative clearance from the Delegation of Public Health, Littoral Region (Re: DD20/L/MSP/DRSPL/CDS) after approval of the protocol. Special consideration was taken to minimize any health risks to the participants and involvement was strictly voluntary. The objectives of the study were explained in detail to each participant who signed a consent form. The participant’s documents were given a code to protect the integrity of the study subjects. At the end of the study, the participants were treated with doxycycline (200mg daily for 28 days).

### 2.2 Study site

**Figure 1:**
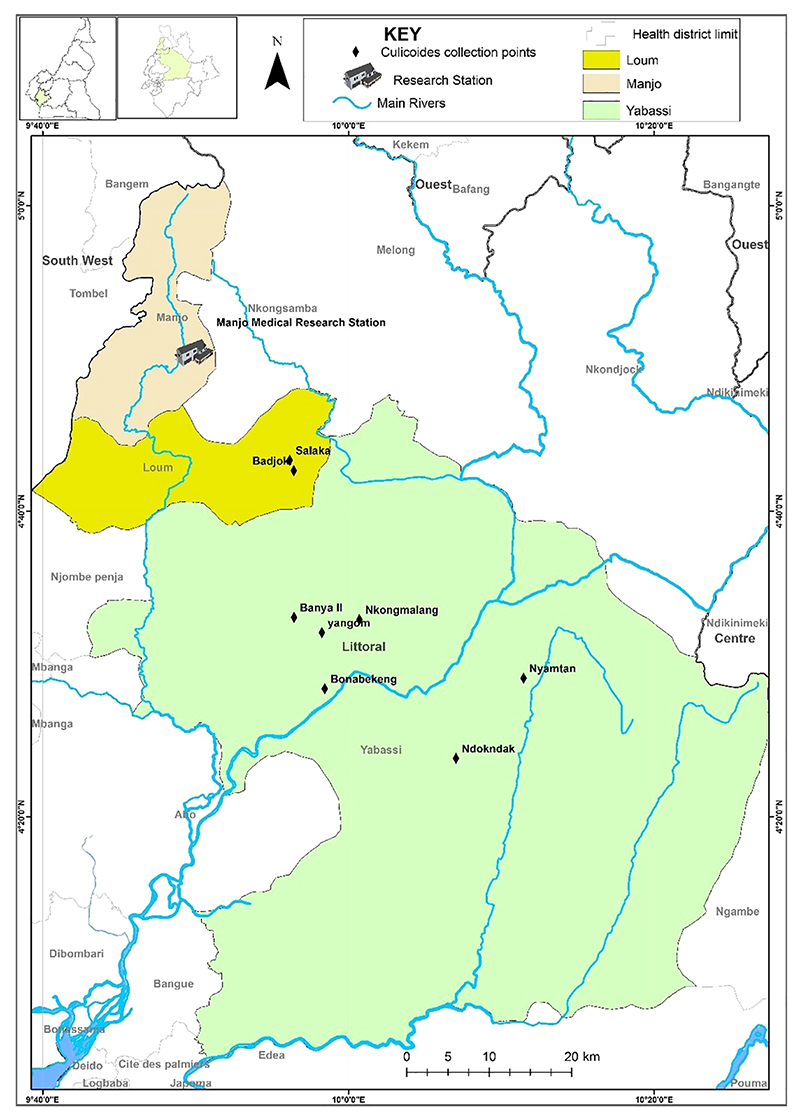
*Culicoides* midge collection sites at Yabassi health districts.

The study was carried out in the Yabassi health district (Yabassi Sub-Division), situated in the Nkam Division in the Littoral Region of Cameroon. The study site in the equatorial rain forest lies between latitudes 4°27’16” N and longitudes 9°58’56” E (Figure 1). It experiences an equatorial climate with a long rainy season (April to November) and a short dry season (December to March) with an average annual temperature of 26.8°C. Moderate temperatures range from 23-25°C and mean annual rainfall of 2-4mm. The average annual relative humidity is 80.8%. The main geographical feature is the Nkam River valley, flanked on both sides by a gently undulating plain with an average altitude of 1000m above sea level. The total population was estimated at 12,000 inhabitants, who are mainly farmers (small cash crop plantations of cocoa, palm oil plantains, bananas) and traders, while some are involved in hunting and fishing activities.

### 2.3 Collection of engorged *Culicoides* species

*Culicoides* species were collected using the drop trap technique (18, 19). Microfilarial (mf)-positive volunteers (≥ 21-year-old males) were assessed by thick blood smear technique. The volunteers (two per session, having ≥520mf/ml of blood) were placed under a rectangular netting cage trap (3x2x2x2m), which was raised for 15- 20 min to allow contact between the host and midges and then lowered to trap the attracted midges. After about 15-20 mins (the expected time for most of the trapped night-biting midges to be fully blood-fed), the engorged *Culicoides* species trapped on the netting materials were gently aspirated by a well-trained fly collector technician with a locally made mechanical aspirator and blown into 50 ml Falcon tubes (Merck KGaA, Darmstadt, Germany), 3/4 filled with plaster of Paris (POP). Using a bright torch light, fully fed midges were separated from (aspirated) non-engorged or partially engorged midges into a different tube and labeled. Fully engorged midges could be seen from the blood-filled reddish abdomen. The POP formed an absorbent layer at the bottom of the tubes to retain moisture. Biting midge collections were performed between 6 p.m. and 6 a.m. and caught midges were maintained in the tubes for 3-5 days in the field as previously described (23). The collected biting midges were fed with 15% sugar solution and then transported to the main research laboratory in Manjo in a cool box for maintenance.

### 2.4 Laboratory maintenance of engorged *Culicoides* species

**Figure 2:**
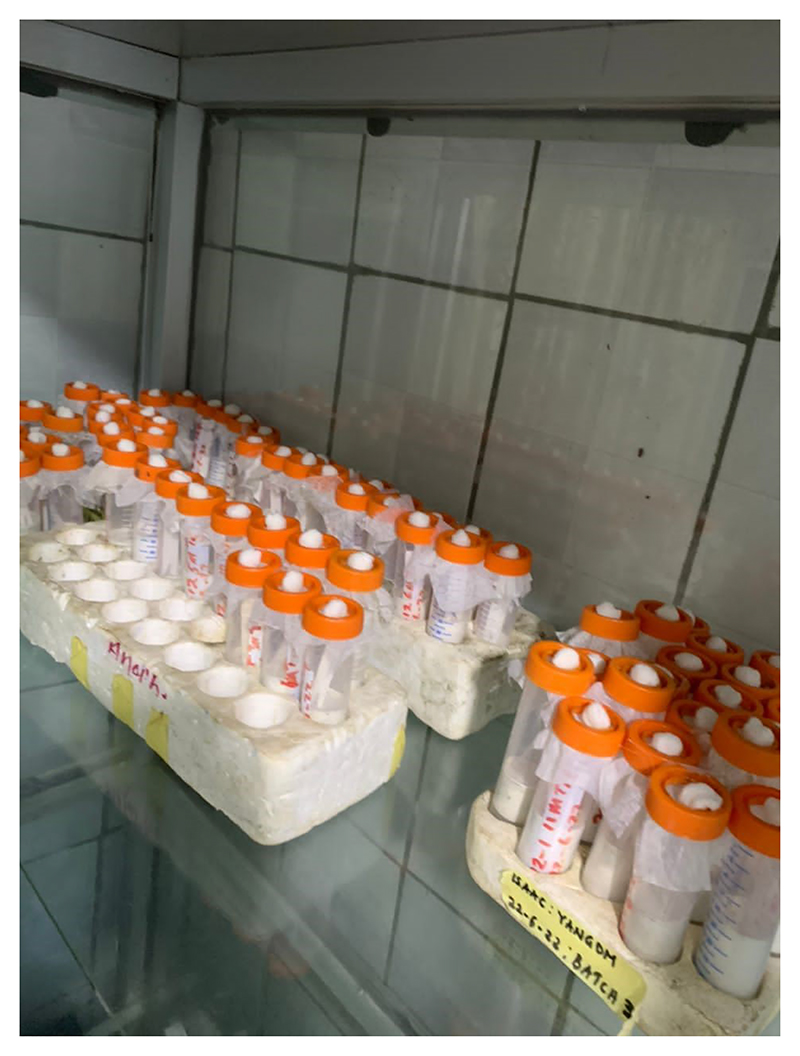
Maintenance of *Culicoides* species in the insectarium at the Manjo laboratory.

*Culicoides* species were maintained at 24°C and relative humidity (RH) of 75 ± 5% in the insectarium at the Manjo laboratory (Figure 2). In short, a hole (0.5 cm in diameter) was made in the lid of each rearing tube to permit ventilation and feeding of the midges in captivity. This hole was covered with fine netting (pore size 0.5 mm) on which the sugar solution-moistened cotton was placed for midge-feeding. Engorged *Culicoides* were fed daily with 15% sugar solution soaked in cotton gauze. Additionally, 3–4 drops of distilled water were added daily using a 10 ml syringe to keep the bottom of the tubes moist. To record midge survival, the rearing tubes were inclined and slightly agitated. Generally, insects have the tendency of moving upward, especially in a closed system. With this in mind, we could easily determine motile and immotile midges. The immotile midges were considered dead after about 10-20 seconds of observation. Dead midges were removed from the tubes daily and stored in 80% alcohol. Mortality of the midges was recorded daily and immobile midges were declared dead after observing them for one minute.

### 2.5 Morphological identification of *Culicoides* species

Morphological identification was done on euthanized engorged *Culicoides* species following the examination of the wing pigmentation pattern and male genitalia under a dissecting microscope using a combination of identification keys (24, 25).

### 2.6 Isolation of *M. perstans* infective larvae

After a total of 12 and 14 days of maintenance in the insectarium, the live *Culicoides* species were knocked down in tween 20-killing solution for 1-2 minutes and rinsed in distilled water for approximately 15-30 seconds. The dead midges were placed on sterile microscope slides containing a drop of the dissecting medium, RPMI-1640 or DMEM (Sigma-Aldrich, Munich, Germany) supplemented with a 2% antibiotic cocktail (penicillin-streptomycin-neomycin; Thermo Fisher Scientific, Schwerte, Germany).

Under the dissecting microscope (Motic, Wetzlar, Germany), the head, thorax and abdomen of the knocked down midges were separated and teased just after morphological identification. This set-up allowed for 1-2 minutes for the larvae to migrate out of the various anatomical regions. All infective larvae (L3) were isolated into sterile dissecting wells labeled head (H), thorax (T) and abdomen (A) containing the same medium.

### 2.7 Data processing and analysis

Data were collected daily on record sheets and entered into a template designed on Microsoft Office Excel 2007 (Microsoft, Redmond, USA). Analyses were performed using R Statistical Language version 4.4.0 (26). Descriptive analysis was conducted to illustrate the number of collected midges per collection season, and per month, as well as the number of live and dead midges dissected, the numbers of recovered L3 from dissected midges per donor and sampling date and *Culicoides* body part (head, thorax, and abdomen). The *ggplot2* (27) package was used for data visualization. A Mann-Kendall Trend test was used to determine whether the trend was monotonic in time series data (mean daily collection of midges at different months, monthly dissection of *C. milnei, C. grahamii* and others). The association between the donor’s load and midge survival was investigated by fitting a Cox proportional hazard regression model (also referred to as Cox regression in this document) with “time to death” as the outcome variable and the donor’s microfilaria load as a predictor variable using the function *coxph* from the package *survival* 3.7-0 (28, 29). The model was then used to determine the predicted survival probability, and visualized with Kaplan Meier curves using the package *survminer* 0.4.9 (30). The number of recovered L3 produced by infected live midges was compared among donors using the Kruskal-Wallis rank sum test, followed by Dunn’s multiple comparison test using rank sums with Bonferroni adjustment when the former test revealed a significant difference (p-value<0.05).

## 3 Results

### 3.1 Engorged *Culicoides* collection

A total of 13,658 engorged *Culicoides* (midges) were collected from February 2022 to January 2023. Five *Culicoides* species were identified namely; *C. milnei, C. grahami, C. inornatipennis, C. fulvithorax and C. neavei* as anthropophilic night biters. The mean parasite load of the six different donors, their respective ages and the period of midge collection are shown in Table 1. Two donors were used per session and the mean parasite load of the donors were quantified every 3 months throughout the study period.

The months with the highest number of days of midge collection were March 2022 and October 2022 with 14 days of collection each, while during the months of December 2022 and January 2023 only 3 days of collection each was performed. In total, 13,658 midges were collected within 112 days between February 2022 and January 2023 (Table 2). In general, two donors per week always participated in the midge collection

### 3.2 Influence of seasons on the abundance of engorged *Culicoides* species

**Figure 3:**
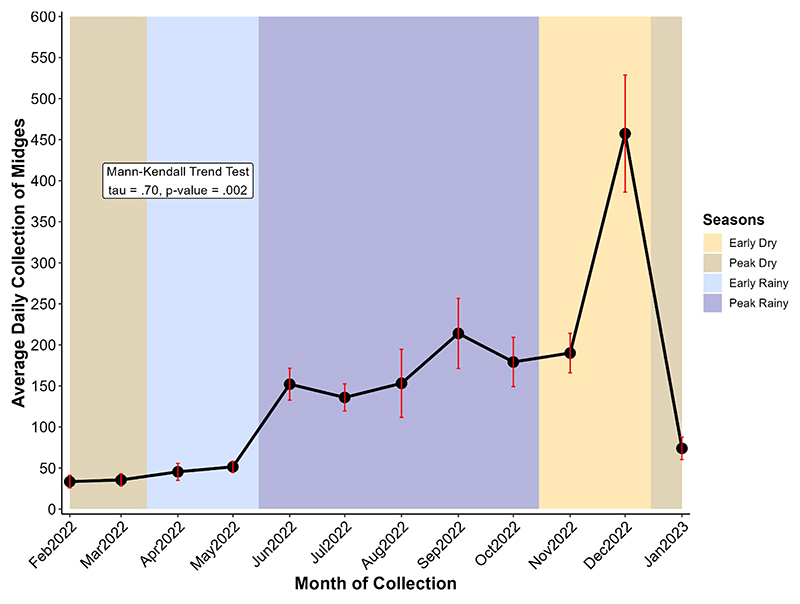
Variation of average daily collection of midges at different months. Midge collection took place from February 2022 to January 2023 and the year is divided into four seasons: the early dry (mid-October to mid-December, ~2 months), the peak dry (mid-December to mid-March, ~3 months), the early rainy (mid-March to mid-May, ~2 months), and the peak rainy (mid-May to mid-October, ~5 months) seasons. Points represent the average and error bars (in red) represent the standard error of the mean.

The average daily collection of engorged *Culicoides* was analyzed by calculating the number of engorged midges collected during a month divided by the number of days of collection of the respective month. In general, we grouped the months into seasons (Figure 3) and differentiated them into the early dry season (mid-October, November, early December), peak dry season (mid-December, January, February, early March), early rainy season (mid-March, April, early May) and peak rainy season (mid-May, June, July, August, September, early October). February to May (early and peak dry season) showed the lowest values of average daily collection of midges with a weak and gradual increase (33.45 in February, 35.57 in March, 45.44 in April, and 51.50 in May). The increase became more evident from June to October (152.17 in June and 179.21 in October) with slight fluctuations (peak rainy season). The peak average daily midge collection was observed in December (457 midges per day), an early dry season month with low or no rainfall and moderate temperatures (Table 2; Figure 3). After the early dry season, we noticed a drastic drop in collection in January which is the peak dry season. A Mann-Kendall trend test revealed the presence of a monotonic trend (p-value = 0.002), that is the direction of variation (increase here) in the daily average collection remained consistent throughout months of collection.

### 3.3 Average daily dissection of *C. milnei* and other species

**Figure 4:**
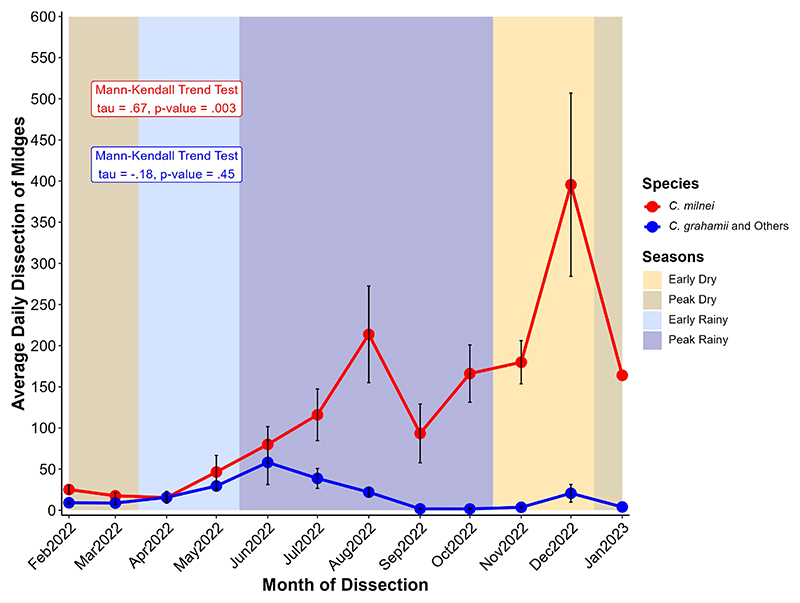
Variation of average daily dissection of midges at different months for *C. milnei* (in red) and other species (in blue). Midge collection took place from February 2022 to January 2023. The year is divided into four seasons: the early dry (mid-October to mid-December, ~2 months), the peak dry (mid-December to mid-March, ~3 months), the early rainy (mid-March to mid-May, ~2 months), and the peak rainy (mid-May to mid-October, ~5 months) seasons. Points represent the average and error bars (in red) represent the standard error of the mean. Points represent the average for the respective species and error bars (in black) represent the standard error of the mean for the associated point.

The average daily dissection of midges was higher throughout the year in *C. milnei* as compared to all other species combined (Figure 4). *C. milnei* displayed a gradual increase in average daily dissection from the peak dry season to the rainy seasons (early and peak) with a drop in September. From the peak rainy season to the early dry season, there is a sharp rise in December (400 midges/day) followed by a drastic drop in January (150 midges per day, the peak dry season. Mann-Kendall trend test revealed a consistent increase in the daily average dissection of midges through months for *C. milnei* (p-value = 0.003), while other species did not demonstrate a monotonic variation (p-value = 0.45). The trendin other species combined is similar, but we noticed a drop in the number of midges dissected per day during the peak rainy season. There was no monotonic evolution (Mann-Kendall trend test, tau = 0.18, p=0.45) of the average daily dissection of *C. grahamii* and other midges combined (Figure 4).

### 3.4 Survival of engorged *Culicoides* midges

#### 3.4.1 Overall survival of engorged *Culicoides* species

**Figure 5:**
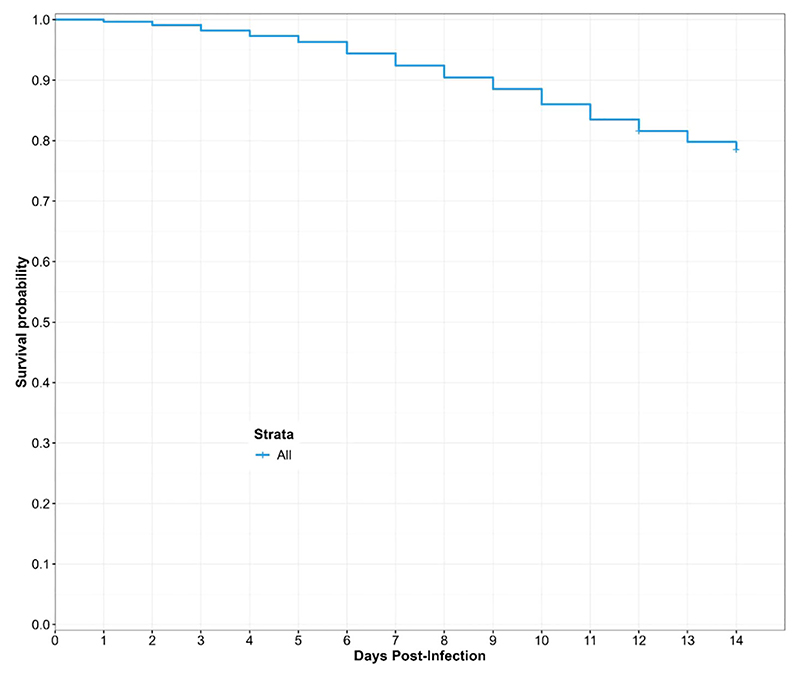
Overall predicted survival probability of engorged *Culicoides* midges during the laboratory rearing. The curve is derived from a Cox Hazard proportional regression model with “time (in days) to death” as the outcome variable and the donor microfilaria load as the predictor variable.

We investigated the survival over the rearing duration (12 and 14 days) of engorged *Culicoides* from the different donors using a Cox proportional hazard regression model. In total, six different donors coded AA-FF participated in the study. We assessed the survival probability of engorged *Culicoides* species 12 days post-infection. At the end of rearing for 12 days post-infection, we obtained some stage two larvae (L2) ( Supplementary Table 1). We extended the rearing period to 14 days to permit the development of L3 larvae. Overall, we recorded a gradual decrease in the predicted survival probability until 78.5% at the end of 14 days of laboratory maintenance (Figure 5).

#### 3.4.2 Influence of the donor microfilaria load on the survival of engorged *Culicoides*

**Figure 6:**
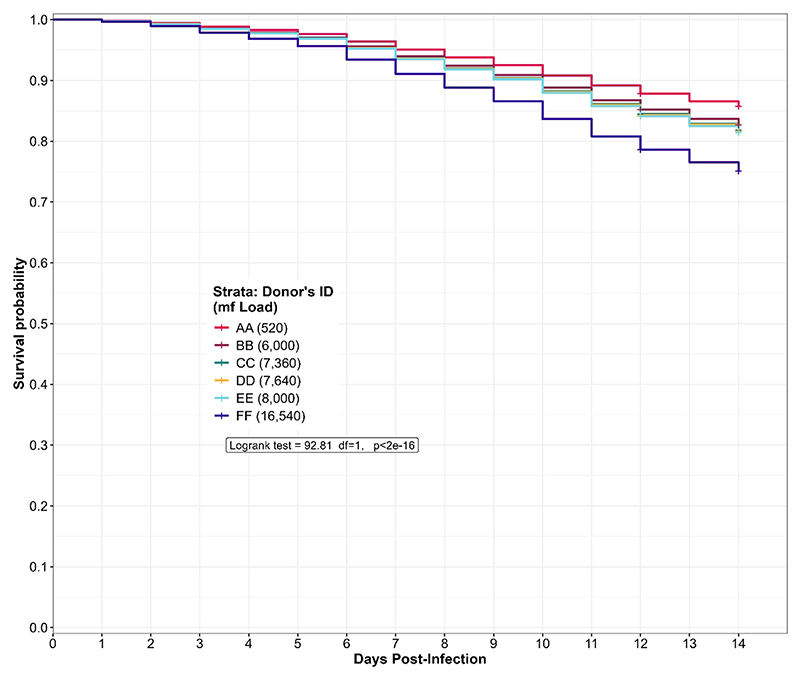
Predicted survival probability of engorged *Culicoides* midges from the different donors during the laboratory rearing. The log-rank test revealed a significant variation (p<2e-16) in the predicted survival probability of the donor microfilaria load. The curve is derived from a Cox Hazard proportional regression model with “time (in days) to death” as the outcome variable and the donor microfilaria load as the predictor variable.

In general, five out of six donors had predicted survival probability of 95% (Figure 6) and above for the first six days of the rearing process. At the end of the study, we observed that midges that fed on donor FF (16,540mf/ml) with the highest microfilaria load had the lowest predicted survival probability when compared with midges that fed on donor AA (520mf/ml) with the lowest microfilaria load. In contrast, midges that fed on donors with intermediate microfilaria loads (between the two extremes) had clustered values and progression of predicted survival. Of note, the predicted survival probability of the *Culidoides* species that fed on the donors EE (8,000 mf/ml) and BB (6,000 mf/ml), were assessed for 12 days post-infection, while the survival probability of biting midges engorged from donors AA, CC, DD and FF was assessed for 14 days post-infection. During the 14-day rearing period, *Culicoides* species which fed on donor AA (520mf/ml) showed the highest (85.7%) predicted survival probability while engorged midges from donor FF (16,540mf/ml) presented the lowest (75.1%) predicted survival probability amongst the four donors (AA, CC, DD, FF) that had the same rearing duration. The biting midges engorged from donor BB (6,000mf/ml) showed a slightly higher (85.2%) survival probability when compared to donor EE (8,000mf/ml; 84.1%) which were both reared for 12 days. There was a statistically significant (log-rank test, p-value<.0.0001) variation in the midges predicted survival probability to donor microfilaria load. Indeed, midges that fed on donors with low microfilaria load survived more under laboratory maintenance (85.7%) than those fed on highly infected donors (75.1%). In other words, the donor microfilaria load was negatively associated with midge survival (Figure 6).

### 3.5 Collection and dissection of engorged alive and dead *Culicoides* species

**Figure 7:**
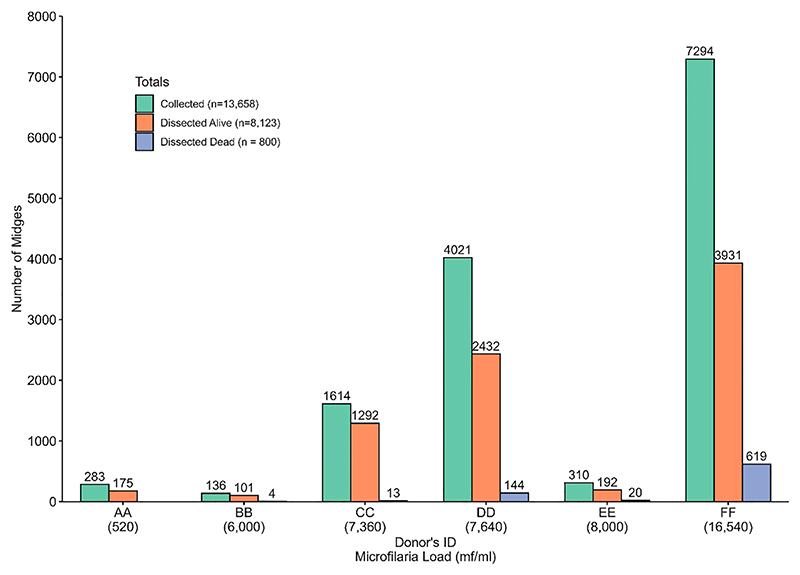
Distribution of collected and dissected (alive and dead) *Culicoides* midges among the different donors.

*Culicoides* collection and isolation of infective larvae from dissected live and dead midges differs between the six donors. To decipher whether a high microfilaria load is associated with dead midges, we dissected dead midges mainly from late July 2022 to January 2023 (dead midges from donor AA were not analyzed). Donor FF (who participated in the study from May 2022 to January 2023) recorded the highest number of engorged midges (n=7,294) caught and dissected both alive (n=3,931) and dead (n=619), followed by donor DD with 4,021 engorged *Culicoides* collected from which 2,432 alive and 144 dead midges were obtained (Figure 7). We collected (136) and dissected (101 live midges and 4 dead midges) the least from donor BB (March to May 2022).

**Figure 8:**
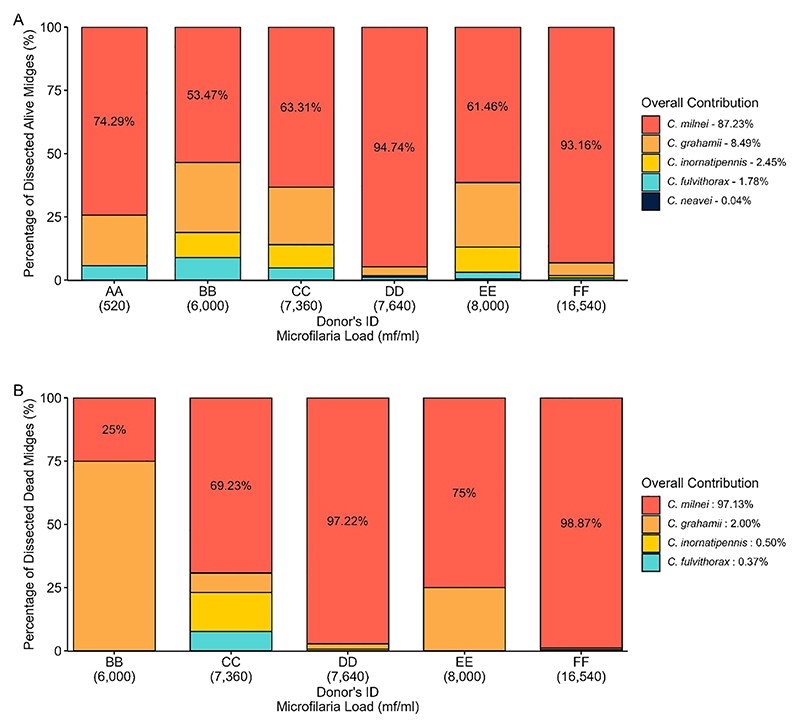
Overview of dissected **(A)** alive and **(B)** dead *Culicoides* species per donor.

Out of the five engorged live *Culicoides* species (*C. milnei, C. grahamii, C. inornatipennis, C. fulvithorax and C. neavei*) identified from the donors after 12 and 14 days of laboratory maintenance, three were common in all six donors but *C. neavei* was only obtained from donor DD and EE (Figure 8A). *C. milnei* was the most abundant species across all the donors accounting for more than 50% of midges identified and dissected for every donor. *C. milnei* accounted the highest for donor DD (94.74%) followed by 93.16% for donor FF for all midges dissected. Overall, *C. milnei* contributed 87.23% of all live midges dissected in this study (Figure 8A). In contrast, only four *Culicoides* species (Figure 8B) were dissected dead from the donors (*C. neavei* absent). *C. milnei* accounted for more than 97.13% of all dead midges dissected of four out of five donors (donor BB, 25%) with the highest from donor FF (98.87%) followed by 97.22% from donor DD.

### 3.6 Contribution of dissected *Culicoides* species to L3 production

**Figure 9:**
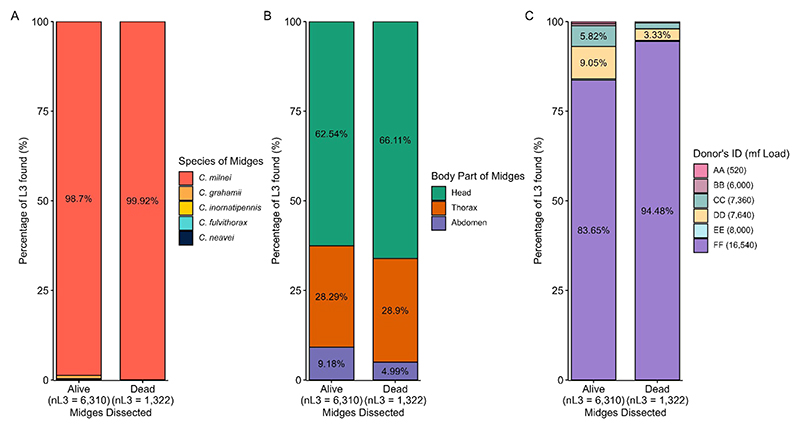
Contribution of dissected *Culicoides* species to L3 production. **(A)** Percentage of L3 found per live and dead *Culicoides* species dissected. **(B)** Percentage of L3 found per dissected midge body part in live and dead midges. **(C)** Percentage of L3 found per donor in live and dead midges.

*C. milnei* accounted for 98.7% (n=6,310 L3) of total L3 recovered, while for dead dissected midges, 99.92% L3 recovery was obtained from the same species (Figure 9A). Throughout the dissection of live and dead midges (Figure 9B), L3 was recovered more from the head (62.54% and 66.11% in live and dead midges, respectively), followed by thorax (28.29% and 28.9% in live and dead midges, respectively) and lastly by abdomen (9.18% and 4.99% in live and dead midges, respectively). The donor FF (16,540mf/ml) with the highest microfilaria load contributed the highest to L3 production (83.65%) followed by donor DD (7,640mf/ml) with 9.05% during dissection of live midges. This was similar to results from the dissection of dead midges in which donor FF still contributed the highest to L3 recovery of 94.48% (Figure 9C).

### 3.7 The distribution of L3 per infected live midges between the different donors

**Figure 10:**
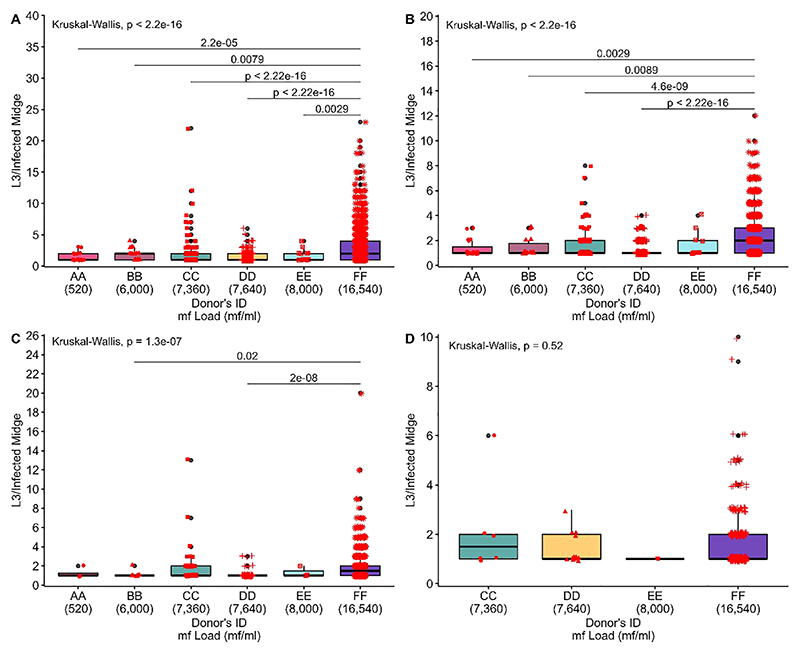
Distribution of L3 per infected live midge. **(A)** The overall distribution of recovered L3 from live midges. **(B)** Distribution of recovered L3 from the head of live midges. **(C)** Distribution of recovered L3 from the thorax of live midges. **(D)** Distribution of recovered L3 from the abdomen of live midges. The number of recovered L3 produced by infected live midges was compared among donors using the Kruskal-Wallis rank sum test, followed by Dunn’s multiple comparisons test using rank sums with Bonferroni adjustment when the former test was significant (p-value<0.05).

The overall L3/infected midges from the head, thorax and abdomen (combined) for donors AA, BB, CC, DD and EE was significantly different for midges that fed on donor FF as compared to all other donors (Kruskal-Wallis, P<2.2e-16, Figure 10A). In the head, a significant difference of recovered L3 from infected midges was found between donor FF and all other donors, except donor EE (Kruskal-Wallis, P<2.2e-16, Figure 10B). The L3 from the thorax of infected midges from various donors varied significantly (Kruskal-Wallis, p=1.3e-07, Figure 10C) and the source of this variation was identified between the pairs BB-FF and DD-FF. The L3/infected midge from the abdomen showed no significant difference (Kruskal-Wallis, p=0.53) amongst the donors. No L3 was recovered from the abdomen of live midges that were fed on donor AA and BB. The highest L3 recovered from infected midges was on donor FF for all anatomical regions (head, thorax and abdomen). An overview of live and dead midges and collected L3 from the different donors and body parts of the midges are shown in Tables 3 and 4, respectively. Moreover, a spreadsheet about individual results from the midges is shown in the Supplementary Material.

### 3.8 Large-scale production of *M. perstans* infective larvae

As depicted in Table 3, out of the 13,658 midges that were collected, a total of 8,123 *Culicoides* survived the rearing and were dissected over a period of 12 months (February 2022-January 2023). In total, we recovered 6,310 L3 from 2,335 *M. perstans*-infected midges, resulting in 2.70 L3 per infected midge. A total of 7,086 dissected *C. milnei* were responsible for 98.7% (6,228 L3) of total L3 recovered. Only 58 L3 were obtained from 690 *C. grahamii* dissections. In addition, 12 L3 each were obtained from 199 C. *inornatipennis* and 145 *C. fulvithorax. C. neavei* (3 collected) were all negative for *M. perstans* L3 (Table 3). In total, 459 L2 and no L1 were obtained during the dissection process ( Supplementary Table 1). The majority of infective larvae were obtained from the head region (n=3,946), followed by thorax (n=1,785) and the abdomen (n=579; Table 3). Moreover, we analyzed dead midges from the donors’ BB, CC, DD, EE, FF (Table 4). In total, 800 dead midges have been analyzed during the collection months of February to April 2022 and June to November 2022. In total 1,322 L3 was recovered from all dead midges but 1,321 have been obtained solely from dead *C. milnei* midges and 1 L3 from *C. grahamii* (Table 4). The graphical representation of various replicates and, detailed numbers about infected dead midges and L3s, dissected dead midges and recovered dead L3 from the anatomical regions are shown in Supplementary Figure 1 and Supplementary Tables 2−4 respectively. From dead midges dissected, we recorded 377 infected dead midges. A total of 1,249 dead L3 were obtained from donor FF, who had a high mircofilaria load. In contrast, only 32 dead L3 was obtained from 73 dead infected midges out of 181 dead midges dissected from donor BB, CC, DD and EE combined, who had lower microfilaria numbers in the periphery.

## 4 Discussion

In this study, we established that *M. perstans* infective larvae can be produced in a large scale from engorged *Culicoides milnei* using a drop trap technique. Important factors for the efficacy of L3 collection were the session of collection, the microfilaria load of the donor, and the duration of laboratory rearing. Indeed, environmental factors play a crucial role in the transmission of filariae species influencing vector transmission dynamic and fitness and consequently, the efficacy of infective larvae transmission (31, 32), but these factors remain uncertain for *M. perstans* transmission. Previously, 13 *Culicoides* spp were identified in Cameroon (24, 25). Here we could show that 5 species (*C. milnei, C. grahamii, C. inornatipennis, C. fulvithorax* and *C. neavei*) fed on the donors. C. *milnei* was the most abundant species collected from which 98.7% (n=6,228) *M. perstans* L3 were obtained, confirming that this midge species is the major vector for *M. perstans* transmission in the South-West and Littoral region of Cameroon (18, 19).

Six microfilaria-positive individuals participated in this study and *Culicoides* midges were collected using the drop trap technique (18, 19). The midges were reared in the insectarium to recover *M. perstans* L3 from the various parts of the midges. We obtained that extension of the laboratory rearing from 12 to 14 days increases the rates of L3 collection. Within the 12 months of the study, we collected 13,658 midges from which 2,328 (17.04%) harbored 6,294 L3, giving a total of 3 L3 per infected midge. A study with wild-caught *Chrysop* flies within a 12 months study period in Gabon obtained 50 L3 per infected fly (33). The differences in the L3 rate per vector can be explained by the large size of the *Chrysop* fly compared to the tiny *Culicoides* midge. Thus, *Chrysop* fly can take up more blood and larvae. In contrast to the lower L3 infection rate per midge, we observed a higher midge survival probability of 78.5% compared to the *Chrysops* survival rate of 43% (33). However, the donor with a high microfilaria load (16,540 mf/ml) had a significantly lower survival probability of engorged midges compared to donors with lower microfilaria loads (520-7,360 mf/ml). These findings confirm our results of higher mortality rates of L3 in *Chrysops* flies that were infected with 100 *Loa loa* mf compared to inoculation of 50 *Loa loa* microfilariae (34). Another study showed that increasing the dose of *Dirofilaria immitis* microfilariae increases the mortality of *Aedes aegypti* (35). These findings highlight that infectious dose could be a major factor affecting the survival of the vectors during laboratory rearing.

Vector mortality is a limiting factor of nature in filarial transmission. It has been observed that all the microfilariae-infected midges cannot survive the period which is required for the development of L3 (36). Indeed, further analysis revealed that the majority of dead infected midges have been obtained from the donor with high microfilaria loads in the peripheral blood, suggesting that increased infection dose led to increased mortality of the midges. Production of L3 requires optimal laboratory rearing conditions but also presupposes a balanced selection of volunteers with moderate to high microfilaria numbers (7,000 − 16,000 mf/ml). These findings have been confirmed by other studies about the *Loa loa* vector mortality since it has been suggested that the emergence of *Loa loa* L3 from the *Chrysops* head incurs a significant amount of excess mortality (37) and that *Loa loa* infection *per se* but also intensity of infection influence vector mortality (38). Similarly, another study showed that the emergence of *Onchocerca volvulus* larvae from the head causes great irritation to the fly, eliciting proboscis ‘milking’ actions associated with stress, and damage to the labio-hypopharyngeal membrane and musculature thereby causing its death and overall high mortality rates of the vector (39). Despite the higher mortality rate of midges from donors with high microfilaria load, we determined that midges surviving to day 14 yielded significantly higher numbers of L3 as compared to midges from donors with low to medium parasite densities, confirming results from *Wuchereria bancrofti* infections (40). In addition, a study using the rodent *Bdellonyssus bacoti* mites showed that an increase of microfilariae did not result in a proportional increase in the number of infective larvae of *Litomosoides galiza* (41).

In regards to the collection and survival of the midges, we observed the lowest collection rate (33 collected midges/day) in February, which is the peak dry season characterized by no rainfall. However, the weather is dry thereby creating a warmer environment for the midges which might reduce their abundance and may have affected their survival before being transported to the laboratory for rearing. The month of December (early dry season) recorded the highest collection of midges (457 collected midges per day), which is characterized by little or no rainfall and moderate temperatures. Moderate temperatures and low rainfall might support vector survival and abundance have been confirmed with the filarial vector *Culex quinquefasciatus*, showing lower vector density in the rainy season in comparison to dry seasons in different endemic areas of the tropics (42). Indeed, environmental factors like temperature, humidity, wind and rainfall have been shown to affect the survival and activity of *Culicoides* midges (43) and it is suggested that midges can better survive in moderate temperatures (44, 45).

Owing to the significance of blood-feeding in the life cycle of the midge, it is an important component for mansonellosis transmission between human and vector hosts. The efficiency of the passage can be influenced by many factors such as the distribution and periodicity of microfilariae in capillaries of hosts for vector infection, blood-feeding duration, and frequency of transmission from vector to vertebrate. Obtaining human blood for insectary colony maintenance and larvae production can be problematic due to several ethical and safety issues. To avoid using humans as donors for engorgement of midges, artificial membrane systems can be used for this purpose. Membrane-feeding assays have been widely used in transmission research and insectary colony maintenance, but human blood as bait is still a limited source since blood banks mainly provide blood for medical emergencies rather than experimental work at insectaries. Thus, different baits could be used to minimize the limitations of using human hosts or human blood. Artificial membrane feeder systems have been established to evaluate topical mosquito repellents (46), transmission-blocking drugs and vaccines (47), and endectocides (48). Using artificial membrane feeding systems instead of human donors resolves the ethical issues concerning human welfare and avoids sophisticated field work required for the drop trap technique. Indeed, artificial membrane feeding systems (49, 50) and murine models for blood feeding (51) have been established for *Culicoides* midges. Therefore, further work should focus on the establishment of an artificial feeding system for *Culicoides* that can be combined with the proposed conditions for large-scale production of L3. We hope that the shown findings encourage laboratories to conduct colony maintenance, vector-parasite interactions, and entomology-related research by seeking inexpensive and effective artificial membrane feeding methods, along with available blood sources that yield the optimum growth and reproductive rates of the vectors of interest.

Finally, we acknowledge two main limitations of the study. Firstly, the collection of *Culicoides* midges was performed for only one year and thus sessional fluctuations over several years can influence vector abundance and biting behavior. Long-term studies need to be performed to confirm the findings. Secondly, the majority of *Culicoides* midges was engorged on the high-load microfilaria donor FF. This donor was readily available and had the longest collection period of engorged midges unlike the other donors leading to a sample bias. Further studies should include donors with different microfilariae loads and collect engorged midges in the early dry session over the same collection time to quantitatively analyze the association of microfilaria load with vector survival and L3 collection.

## 5 Conclusion

In this study, we implemented a procedure to collect and maintain engorged midges to scale up the production of *M. perstans* infective larvae. Furthermore, we showed that the early dry season with little or no rainfall and moderate temperatures are optimal for the collection and laboratory maintenance of the midges. We suggest that production of high yield *M. perstans* L3 requires a mixed set of moderate and high microfilaria donors. Compliance to these multifactorial conditions allows large-scale production of *M. perstans* L3, which are urgently needed to implement *in vitro* culture systems for drug testing approaches and the establishment of *M. perstans* animal infection models.

## Supplementary Material

Supplementary Material

## Figures and Tables

**Table 1:** Characteristics of the six donors and months of participation.

Donor’s code	Age (years)	Mean parasite load (mf/ml)	Months of collection
AA	65	520	July 2022
BB	68	6,000	March-May 2022
CC	50	7,360	February-June 2022
DD	49	7,640	July 2022 - January 2023
EE	48	8,000	February-April 2022
FF	60	16,540	May 2022 - January 2023

**Table 2:** Overview of period of collection and number of collected biting midges.

Months	Period (days) of collection	Total days of collection per month	Number of collected midges	Number of collected midges/day
February 2022	01-03	11	368	33.45
	09-11			
	15-18			
	21-23			
March 2022	01-03	14	498	35.57
	07-09			
	14-16			
	21-23			
	28-30			
April 2022	03-05	9	618	45.44
	18-20			
	25-27			
May 2022	02-04	12	515	51.50
	09-11			
	16-18			
	23-25			
June 2022	06-08	12	1,826	152.17
	13-15			
	20-22			
	27-29			
July 2022	04-06	9	1,224	136.00
	18-20			
	25-27			
August 2022	01-03	9	1,379	153.22
	08-10			
	15-17			
	22-24			
	28-30			
September 2022	12-14	8	1,712	214.00
	19-21			
	26-28			
October 2022	03-05	14	2,509	179.21
	10-12			
	17-19			
	24-26			
	31			
November 2022	01-02	8	1,521	190.12
	21-23			
	28-30			
December 2022	05-07	3	1,372	457.33
January 2023	11-13	3	222	74.00
**TOTAL**		**112**	**13,658**	**1,722**

**Table 3:** Overview of live *Culicoides* spp and isolated *M. perstans* infective larvae from the various parts of the midges.

Donor’code (mf/ml)	No. of collected midges	No. of dissected alive midges	No. of dissected midge species					No. of L3 recovered from dissected *Culicoides* species															No. of inf. midges	Total no. of L3	No. of L3 per inf. midges
			*C. milnei*	*C. grahamii*	*C. inornatipennis*	*C. fulvithorax*	*C. neavei*	*C. milnei*			*C. grahamii*			*C. inornatipennis*			*C. fulvithorax*			*C. neavei*					
								H	T	A	H	T	A	H	T	A	H	T	A	H	T	A			
AA (520)	283	175	130	35	0	10	0	26	5	0	2	0	0	0	0	0	0	0	0	0	0	0	24	33	1.38
BB (6,000)	136	101	54	28	10	9	0	26	10	0	0	0	0	0	1	0	0	0	0	0	0	0	21	37	1.76
CC (7,360)	1,614	1,292	818	293	119	62	0	236	95	13	15	2	0	2	2	0	0	2	0	0	0	0	191	367	1.92
DD (7,640)	4,021	2,432	2,304	87	12	27	2	479	71	18	2	0	0	1	0	0	0	0	0	0	0	0	414	571	1.38
EE (8,000)	310	192	118	49	19	5	1	18	4	2	0	0	0	0	0	0	0	0	0	0	0	0	15	24	1.60
FF (16,540)	7,294	3,931	3,662	198	39	32	0	3,104	1,577	544	27	8	2	4	2	0	4	6	0	0	0	0	1,670	5,278	3.16
overall	13,658	8,123	7,086	690	199	145	3	3,889	1,762	577	46	10	2	7	5	0	4	8	0	0	0	0	2,335	6,310	2.70

No., number; mf, microfilarial; inf., infected; H, Head; T, Thorax; A, Abdomen).

**Table 4:** Overview of dead *Culicoides* spp. and isolated *M. perstans* infective larvae from the donors BB, CC, DD, EE and FF during collection months February to April 2022 and June to November 2022 (No., number; H, Head; T, Thorax; A, Abdomen).

Donor’s code (mf/ml)	No. of dissected dead midges	No. of dissected dead midge species				No. of L3 recovered from dissected *Culicoidesi* species												No. of inf. midges	Total no. of L3	No. of L3 per inf. midges
						*C. milnei*			*C. grahamii*			*C. inornatipennis*			*C. fulvithorax*					
		*C. milnei*	*C. grahamii*	*C. inornatipennis*	*C. fulvithorax*	H	T	A	H	T	A	H	T	A	H	T	A			
BB (6,000)	4	1	3	0	0	4	0	0	0	0	0	0	0	0	0	0	0	1	4	4.00
CC (7,360)	13	9	1	2	1	14	8	0	0	0	0	0	0	0	0	0	0	7	22	3.14
DD (7640)	144	140	3	1	0	33	11	0	0	0	0	0	0	0	0	0	0	22	44	2.00
EE (8,000)	20	15	5	0	0	3	0	0	0	0	0	0	0	0	0	0	0	2	3	1.50
FF (16,540)	619	612	4	1	2	820	363	65	0	0	1	0	0	0	0	0	0	345	1249	3.62
overall	800	777	16	4	3	874	382	65	0	0	1	0	0	0	0	0	0	377	1,322	3.51

## Data Availability

The original contributions presented in the study are included in the article/Supplementary Material, further inquiries can be directed to the corresponding author/s.
