# Supplementary material for: Large-scale production of Mansonella perstans infective larvae from engorged Culicoides milnei
Source: Front Trop Dis. Author manuscript; Available in PMC 2025 Jan 14. (PMC7617305; doi:10.3389/fitd.2024.1391823)
Supplement: Supplementary Material [file EMS202155-supplement-Supplementary_Material.pdf]

10.3389/fitd.2024.1391823

′

Supplementary material

The Supplementary Material for this article can be found online at: https://www.frontiersin.org/articles/10.3389/fitd.2024.1391823/full#supplementary-material
